# Time Spent Outdoors and Associations with Sleep, Optimism, Happiness and Health before and during the COVID-19 Pandemic in Austria

**DOI:** 10.3390/clockssleep5030027

**Published:** 2023-06-25

**Authors:** Simon Schamilow, Isabel Santonja, Jakob Weitzer, Susanne Strohmaier, Gerhard Klösch, Stefan Seidel, Eva Schernhammer, Kyriaki Papantoniou

**Affiliations:** 1 Department of Epidemiology, Center for Public Health, Medical University of Vienna, 1090 Vienna, Austria; simon.schamilow@gesundheitsverbund.at (S.S.); isabel.santonja@meduniwien.ac.at (I.S.); weitzerjakob@gmail.com (J.W.); susanne.strohmaier@meduniwien.ac.at (S.S.); eva.schernhammer@meduniwien.ac.at (E.S.); 2 Department of Health Promotion and Prevention, Federal Ministry of the Republic of Austria for Social Affairs, Health, Care and Consumer Protection, 1030 Vienna, Austria; 3 Department of Neurology, Medical University of Vienna, 1090 Vienna, Austria; gerhard.kloesch@meduniwien.ac.at (G.K.); stefan.seidel@meduniwien.ac.at (S.S.); 4 Channing Division of Network Medicine, Department of Medicine, Brigham and Women’s Hospital and Harvard Medical School, Boston, MA 02115, USA; 5 Department of Epidemiology, Harvard T.H. Chan School of Public Health, Boston, MA 02115, USA

**Keywords:** time spent outdoors, daylight, sleep, chronic insomnia, chronotype, optimism

## Abstract

Social restriction measures (SRM) implemented during the COVID-19 pandemic led to a reduction in time spent outdoors (TSO). The aim of this study was to describe TSO and evaluate its association with sleep outcomes, optimism, happiness and health-status before and during SRM. Two online surveys were conducted in 2017 (N = 1004) and 2020, during SRM (N = 1010), in samples representative of the age, sex and region of the Austrian population. Information on the duration of TSO, sleep, optimism, happiness and health-status was collected. Multivariable-adjusted logistic regression models were used to study the association of TSO with chronic insomnia, short sleep, late chronotype, optimism, happiness and self-rated health-status. The mean TSO was 3.6 h (SD: 2.18) in 2017 and 2.6 h (SD: 1.87) during times of SRM. Men and participants who were older, married or in a partnership and lived in a rural area reported longer TSO. Participants who spent less time outdoors were more likely to report short sleep or a late chronotype in both surveys and, in 2020, also chronic insomnia. Less TSO was associated with lower happiness and optimism levels and poor health-status. Our findings suggest that TSO may be a protective factor for sleep, mood and health, particularly during stressful and uncertain times.

## 1. Introduction

Biological circadian rhythms are found in every mammalian cell and regulate physiological functions such as sleep, alertness and metabolism. Endogenous circadian clocks adapt to environmental changes, with (day)light being the most potent synchronizer [1]. The key mediator between environmental light conditions and circadian clocks is melatonin, a hormone produced by the pineal gland during nighttime darkness and suppressed by light at night (LAN) [2]. Melatonin secretion is associated with an increase in sleep propensity and is considered a sleep promoting factor in humans. Furthermore, melatonin affects nearly all physiological systems, including the cardiovascular, immunological and endocrine systems [3]. The proper alignment of the circadian clocks with natural light–dark cycles is vital for humans and other mammals, as circadian disruption—an umbrella term for different types of disturbances of the biological clock [4]—may lead to negative health outcomes including cardiometabolic, psychiatric and neurological diseases and immunological malfunction, as shown in experimental and epidemiological studies [5,6,7,8,9,10,11,12,13]. Exposure to daylight can help to realign disrupted circadian rhythms, and sufficient daylight has been linked to beneficial health outcomes. For example, several large-scale epidemiological studies, RCTs and systematic reviews have described the protective effect of time spent outdoors (TSO) on myopia [14,15,16,17]. Moreover, individuals spending more than 30 min outdoors in a day had a lower BMI and were less likely to report type 2 diabetes or cancer in a US-based study [18]. These effects were reduced, but still present, after adjusting for physical activity. However, for other health outcomes, such as sleep and mood outcomes, the existing body of evidence is smaller. Recent evidence from a large cross-sectional and longitudinal study (UK-Biobank, n = 502,000) suggests that increased daylight exposure is linked to lower odds of low mood, lifetime major depressive disorder and use of antidepressants, as well as a greater ease of getting up in the morning, less frequent tiredness, earlier chronotype, and fewer insomnia symptoms [19]. So far, data on the interrelation of the effects between TSO, mood and sleep outcomes are scarce. However, previous analyses of our survey data showed a significant association of optimism with lower odds of chronic insomnia [20], providing a solid basis to further explore the links between TSO, optimism and chronic insomnia.

The rise of the worldwide COVID-19 pandemic and its associated social restrictions measures (SRM) led to robust changes in our daily routines and lifestyle, including reductions in physical activity levels and TSO [21,22,23]. Several studies have described SRM-related changes in sleep duration and sleep quality [24,25,26]. A survey conducted in several countries across the globe, the Global Chrono Corona Survey (GCCS), showed that decreased TSO during SRM was associated with negative changes to sleep, quality of life, physical activity, and screen time [22]. The literature also suggests that low TSO may have negative consequences for mood outcomes such as happiness, but few studies have evaluated this question in the context of the pandemic and social restriction measures. One study in the US showed that compared to participants who reduced TSO during the pandemic, subjects who increased or maintained TSO had lower stress levels and better mental health [27]. Another study conducted in Austria [21] showed that TSO was associated with lower depression and anxiety levels and better mental health outcomes; however, this study was conducted in a convenience sample, with a young population and a majority of women. It remains unclear if these effects can also be observed in other population groups.

In this study, our first aim was to describe the patterns of TSO and evaluate the association of TSO with chronic insomnia, short sleep duration and chronotype in two independent representative samples of the adult Austrian population before (2017) and during the first COVID-19 wave and subsequent SRM in Austria (2020). The second aim was to evaluate the association of TSO with optimism, happiness and self-rated health status. We hypothesized that higher TSO is associated with lower odds of chronic insomnia, short sleep, late chronotype, low optimism, low happiness and bad health status.

## 2. Results

Two online surveys were conducted in Austria, in September 2017 (N = 1004 participants) and in June 2020 (N = 1010), during the first Austrian COVID-19 mitigation period (from 16 March 2020 to 1 May 2020). The two survey samples were taken independently, representative of the age, sex, and country distribution of the adult Austrian general population (18–65 years). Both surveys collected information on sleep habits, lifestyle and sociodemographic characteristics.

In both surveys, participants were distributed evenly across sex categories. The median age of participants in Survey 1 was 43 years of age (IQR: 22), and in Survey 2 between 40 and 44 years (Q1: 30–34, Q3: 50–54). In the exploratory analysis, selected sociodemographic and lifestyle characteristics based on prior knowledge and availability of information were evaluated in relation to increasing levels of TSO in pre-pandemic (2017) and pandemic (2020) times. Detailed characteristics according to selected quintiles (TSOq1, TSOq3 and TSOq5) of the two study samples are shown in Table 1. Older age, male sex, being married or in a partnership, living in a rural area (versus urban area) and having higher levels of physical activity were linked to longer TSO in both study populations. 

Differences in TSO, physical activity and other factors between 2017 and 2020 are shown in Table 2. We observed a reduction in TSO in 2020, with participants spending on average 3.56 h outdoors (SD = 2.18; median = 3.1 h) in 2017 and 2.57 h (SD = 1.87, median = 2.1 h) in 2020. This reduction was observed in men [2017: mean (SD) TSO = 3.70 (2.33) h; median = 3.14 h; 2020: mean (SD) TSO = 2.62 (1.94); median = 2.14] and women [2017: mean (SD) TSO = 3.41 (2.02) h; median = 3.07; 2020: mean (SD) TSO = 2.51 (1.81) h; median = 2.28 h]. Moreover, in 2020, the prevalence of chronic insomnia and late chronotype increased compared to 2017, while the prevalence of short sleep duration decreased. On the other hand, happiness and optimism scores slightly decreased during the pandemic as compared to before. 

We examined the association of daily average TSO with chronic insomnia, short sleep and late chronotype prevalence using GAM splines and found no evidence for a significant departure from linearity (p-for-gain for all outcomes > 0.05) (Supplemental Figure S1). Age- and multi-variable-adjusted logistic regression models for TSO (continuous) and sleep outcomes in 2017 and 2020 are presented in Table 3; the models using quintiles categories as exposure groups are presented in Supplemental Tables S1 and S2 for 2017 and 2020, respectively. In 2017, lower levels of TSO were significantly associated with short sleep [MV-adjusted OR (95% CI) = 0.88 (0.80–0.96)] and late chronotype [MV-adjusted OR (95% CI) = 0.87 (0.80–0.95)], but not with chronic insomnia, except for TSOq2 (vs. TSOq1: MV-OR (95% CI) = 0.45; 0.22–0.98). In 2020, we found a significant association of lower levels of TSO with increased risk of chronic insomnia, short sleep and late chronotype. For every additional hour spent outdoors, the odds of chronic insomnia decreased by 18% [MV-OR (95% CI) = 0.82 (0.71–0.94)] after adjusting for potential confounders. Additionally, adjusting for (a) happiness and optimism, (b) physical activity levels or (c) health status had no effect or only slightly attenuating effects on the association of TSO with sleep outcomes. Similar results were found when modelling quintiles of TSO and sleep outcomes in 2020 (Supplemental Table S2).

Similarly, we did not observe a departure from linearity in the association between TSO and happiness, optimism and health status (Supplemental Figure S2). Table 4 shows age- and MV-adjusted models of the association of TSO with optimism, happiness and health status in 2017 and 2020. Lower levels of TSO were significantly associated with being the least happy, the least optimistic and reporting a poor health status in MV-adjusted models in 2017. The results obtained using TSO quintiles as an outcome were similar to those using the continuous TSO variable (Supplemental Table S3). In 2020 we found similar trends, except for the outcome of least happy, for which the risk estimates for the continuous TSO were non-significant. Nonetheless, for this outcome we observed significant odds reductions for TSOq4–5 compared to TSOq1 in the age- and in the multivariable-adjusted model, which were not significant after additionally adjusting for (a) optimism, (b) physical activity or (c) health status (Supplemental Table S4). Stratified analyses showed no significant variation of the effects across strata of sex on most of the outcomes in both surveys (Supplemental Table S5). 

## 3. Discussion

In this study, based on two independent representative samples of the Austrian population, we found that TSO decreased 1.0 h during the first COVID-19 mitigation measures in 2020 (average daily TSO: median = 2.1 h) compared to previous levels in 2017 (average daily TSO: median = 3.1 h). In both surveys, participants reporting longer TSO were significantly less likely to report short sleep duration and late chronotype and, in 2020, longer TSO was associated with lower chronic insomnia risk [MV-OR (95% CI) = 0.82 (0.71–0.94)]. Furthermore, TSO was also associated with higher levels of optimism and happiness, and a better self-rated health status. 

### 3.1. Changes in TSO during COVID-19 Social Restriction Measures (SRM) and Predictors of TSO

Worldwide, there was a decline in TSO following COVID-19 mitigation measures, as reported in Korman, Tkachev [22]. Haider and Smith [21] also described a reduction in TSO in Austria during SRM; however, they reported shorter TSO in their sample (120 min before and 60 min during the first lockdown). This difference could be due to the fact that this was a convenience sample, collected through social media and other channels, and mainly composed of women and young adults (mean age 36.0 years). Thus, this study population may not be comparable to our samples, which were representative of the Austrian population and, therefore, were overall older and had a higher proportion of men. By contrast, a study [28] in UK office workers found a significant increase in TSO during the 2020 lockdown; the authors explain this surprising result by the flexibility of homeworking and extremely sunny weather in spring 2020. 

Results from our descriptive analysis showed that male sex was associated with higher TSO in the 2017 and 2020 samples. This was in line with results from a Scotland-based study, which showed that men are more likely to spend time in green spaces than women [29], and a study among Chinese students [30], in which men spent more time doing activities outdoors than women. Interestingly, during the COVID-19-SRM (2020), the TSO sex gap declined markedly, possibly due to the suspension of many outdoor work activities, such as construction work, which are traditionally performed by men. In addition, part of the reduction in TSO could be explained by a reduction in commuting times, since during SRM telework was widely implemented, and this might have affected more men than women, since employment rates in Austria are higher among men [31]. Older age also correlated positively with TSO in both surveys, as was also reported among UK-Biobank study participants [32]. Additionally, we found that being married or in a relationship was linked to more TSO, both before and during the pandemic. Furthermore, physical activity (including walking) predicted TSO consistently in both samples, although the questionnaires used for our study did not differentiate between indoor and outdoor physical activity. This association has been described previously [13,18], and provides an interesting opportunity to increase TSO through physical activity and positively impact health via both mechanisms. Participants living in rural areas reported spending more time outdoors than those living in urban areas, in both of our surveys. Spending time outdoors in a natural environment can improve health and wellbeing [33], and those living in cities spend more time doing physical activities if they live near urban green spaces [34]. Additionally, a longitudinal study conducted during COVID-19 stay-at-home orders (n = 20,012) found that spending time outdoors in nature and undertaking physical activity both significantly reduced depression and anxiety scores. The effects were slightly stronger for younger and female participants [35]. Altogether, these results underscore the importance of innovative green city planning, with more parks and green areas and less concrete. 

### 3.2. TSO and Sleep Outcomes

Before and during the COVID-19 pandemic, an increase in TSO was paired with a reduction in the odds of reporting short sleep duration and late chronotype. In 2020, higher levels of TSO were also associated with lower chronic insomnia risk. Similar results were found when comparing quintiles of TSO in relation to most sleep outcomes. These effects were independent of known sociodemographic and lifestyle confounders.

Our 2020 findings were in line with a recently published study [19] which found that an hour increase in TSO is associated with lower odds of suffering from insomnia symptoms. Leger and Bayon [36] have also shown that workers unexposed to daylight were more likely to report insomnia, non-restorative sleep and daytime sleepiness compared to workers exposed to light. Similarly, a study among office workers showed that those working in an environment with daylight reported better sleep quality than those working in windowless offices [37]. However, in our 2017 sample we did not find an association between TSO and chronic insomnia. There might be a few explanations for this discrepancy. First, the reason could be the rather small size of our samples in combination with the low prevalence of chronic insomnia; other studies had notably larger study samples [19,36]. Another explanation could be that TSO was “used” more consciously during the stressful and uncertain SRM period in 2020, which may have resulted in more beneficial effects of TSO on sleep and mental health outcomes compared to pre-pandemic times. 

The association of longer TSO with an earlier chronotype has been reported before by Korman et al. [22], who suggested that bedtimes were delayed during SRM due to increased light exposure in the evening. In this context, TSO during daytime may have counteracted circadian disruptions caused by later sleep times, higher light exposure at night and increased screen time use, improving sleep, and helping to keep circadian rhythms in sync. Before SRM, an older epidemiological study conducted by Rönneberg et al. [38] described the connection between time spent outdoors and chronotype, showing that each additional hour spent outdoors advanced the onset of sleep for almost 30 min. Experimental studies in mice [39] and mathematical models [40] provide solid evidence for the beneficial effects of (day)light exposure on circadian rhythms found in epidemiological studies. Although we did not analyze sleep timings, our results using self-reported chronotypes are consistent with previous findings. 

On the other hand, exposure to artificial light at night (ALAN) has been shown to have detrimental effects on human sleep and health. During the last two decades, an increasing use of ALAN and screens, especially before bedtime, has been observed. Exposure to ALAN in the evening is associated with melatonin suppression [41], a higher prevalence of sleep problems [42] and shorter sleep duration [43]. Sleep problems (e.g., poor sleep quality, insomnia symptoms) and irregular sleep duration (e.g., short or long sleep) have been associated with a higher risk for all-cause mortality, cardiometabolic diseases and cancer [44,45,46,47,48]. Additionally, in adolescents, higher levels of outdoors ALAN correlated with later chronotypes [49]. Conditions mimicking daily sunlight exposure earlier in the day can help advance the circadian phase, and late-evening exposure to blue light is associated with melatonin suppression and circadian phase delay [50]. Furthermore, circadian disruption resulting from high exposure to ALAN has been shown to negatively affect metabolism [51] and the risk of obesity [52]. In contrast, (morning) daylight exposure is associated with beneficial health effects such as less sleepiness during the day, positive metabolic profiles [53], an increased cortisol awakening response [54], higher levels of serotonin and vitamin D, an earlier onset of nocturnal melatonin production [55] and therapeutic effects on sleep [56] and mood disorders [57]. Several properties of daylight exposure, such as wavelength and timing of daylight exposure, can modulate the effects of ALAN on the circadian system. A recently published paper by a broad joint taskforce provided recommendations for daytime, evening, and nighttime indoor light exposure to “best support physiology, sleep and wakefulness in healthy adults” [58]. Animal [59] and human [60] studies show that the melatonin-suppressing effects of light exposure at night are attenuated by the amount and quality of daylight, in particular morning light exposure. Therefore, higher daylight exposure may help mitigate the negative effects of light at night on sleep, mood and health.

### 3.3. TSO and Optimism, Happiness and Health-Status

In addition to sleep and circadian outcomes, TSO was also associated with optimism, happiness and self-reported health status in the two samples analyzed. One study found comparable results for happiness, but did not assess the effects of TSO on optimism [19]. Another online survey from Austria reported that being outdoors >60 min/day was associated with better mental well-being and a lower chance for having depressive symptoms [21]. The association between daylight exposure and common mental disorders, in particular depression, has been studied before. Daytime light therapy alleviates depressive symptoms [61,62] by correcting misaligned circadian rhythms, which is a common feature of major depressive disorders and has been shown to correlate with the severity of depressive symptoms [63]. Furthermore, studies show that intrinsically photosensitive retinal ganglion cells, which primarily communicate light responses to the suprachiasmatic nucleus, also project to other brain areas such as the amygdala and habenula [64], which play a crucial role in emotionality and the development of depressive symptoms [65]. Moreover, daylight exposure enables vitamin D synthesis, which has a protective effect on depression risk [66]. Weitzer et al. [67] showed that dispositional optimism (at the same scale used as in our study) is a protective factor for depression. Participants with longer TSO might tend to be more optimistic, which in turn may serve as a coping mechanism contributing to higher resilience against mood and mental problems, especially in stressful and uncertain times such as the ones experienced during COVID-19-related SRM. 

Our findings on TSO and poor health status are in line with a previously published cross-sectional study [18] showing that TSO is associated with lower chronic disease risk, and the relationship was partly explained by physical activity. Time spent outdoors might result in more physical activity, and several previous studies have reported a protective effect of physical activity on sleep, depression, loneliness and anxiety [68,69]. Acute and regular physical exercise may have beneficial effects on various sleep properties [70], potentially explained by increased energy consumption, endorphin secretion, growth hormone secretion, changes in cytokine concentration and body temperature, improved fitness and changes in body composition [70,71]. Furthermore, aerobic exercise training improved sleep and immunological function and decreased stress hormones levels in patients with chronic primary insomnia [72]. In our analysis, adjusting for walking, moderate or vigorous physical activity only marginally changed the results, suggesting an independent effect of TSO or daylight exposure on sleep, mood and health outcomes. Furthermore, our previous research showed that optimism was a significant predictor of sleep problems, with less optimistic participants having higher odds of chronic insomnia [20]. However, we did not find evidence for confounding by optimism, happiness or physical activity. Thus, it is still possible that optimism acts as mediator of the association of TSO and sleep, or that TSO explains the link between optimism and sleep outcomes. Prospective cohort studies are needed to better understand the complex effects, directionality and interrelations of TSO and physical activity on sleep, mood and health outcomes.

### 3.4. Limitations and Strengths

Our study has a few limitations. First, TSO was self-reported and used as a proxy for daylight exposure, instead of objective light measurements. Second, our association analyses were cross-sectional and, thus, we cannot comment on the direction of the observed associations, and reverse causation may partly explain our study results. Third, although our study samples were large, some of the stratified analyses were underpowered for the less common outcomes (e.g., chronic insomnia). Nonetheless, our study also has several strengths. Detailed sleep information allowed the assessment of chronic insomnia according to four criteria of the International Classification of Sleep Disorders. In addition, our analyses included data from two large, nationwide online surveys in representative samples of the Austrian population. Moreover, exposure information was available for workdays and days off, and potential daily variation was accounted for in the computed weighted average TSO. Finally, the logistic regression models were carefully adjusted for a wide range of potential confounders.

## 4. Materials and Methods

### 4.1. Study Design and Setting

A cross-sectional online survey was conducted in September 2017 among 1004 participants who represented the age, sex and country distribution of the adult Austrian general population (18–65 years). The survey contained 63 questions and focused on detailed information on sleep habits, daily lifestyle and other sociodemographic characteristics. In June 2020, an updated version of the survey was conducted among a newly selected sample of 1010 Austrians, also representative of the adult Austrian general population. New questions were added regarding sleep changes, lifestyle and sociodemographic factors such as employment and working from home during the first Austrian COVID-19 mitigation period (from 16 March 2020 to 1 May 2020). The first Austrian mitigation policy lasted for 50 days, from 16 of March 2020 till 1 of May, and comprised a ban on using public spaces with five exceptions. These were (1) averting an immediate danger to life, limb and property; (2) providing assistance to people in need of support, as well as fulfilling family obligations; (3) covering the necessary basic needs of daily life; (4) fulfilling professional purposes, if necessary; and (5) spending time outdoors for physical and mental relaxation. Both surveys took approximately 30 min to complete and were implemented by Interrogare GmbH, a Germany-based market research institute. The participants took part anonymously and voluntarily. Informed consent was implied through participation. The studies were exempt from Institutional Review Board approval according to Federal Regulation 45 CFR 46.10(b).

### 4.2. TSO Assessment

In both surveys, participants were asked how much time (in hours and minutes) they usually spent outdoors in the light (without a roof over the head) on a work-/weekday and on a day off/weekend. In 2017, different questions covered TSO during spring/summer and during autumn/winter, whilst in 2020 participants were only asked about TSO since the introduction of SRM on 16th March (coincides with early spring in Austria). For 2017, we found a positive linear correlation (r^2^ = 0.4713; *p* < 0.000) between TSO in spring/summer and TSO in autumn/winter, and thus for comparability between the two surveys only results for TSO spring/summer analyses were presented. Using this information, the daily average TSO for each survey (TSO-2017 and TSO-2020, continuous variables) was calculated using the formula “(5*TSO on a weekday + 2*TSO on weekend)/7”. Additionally, we defined an exposure categorical variable based on the quintiles (TSOq1–5) of the distribution of average TSO in each survey.

### 4.3. Outcomes Assessment

Primary outcomes were chronic insomnia, sleep duration and chronotype. Chronic insomnia was defined by the presence of four criteria according to the International Classification of Sleep Disorders, 3rd edition: (a) report of difficulty initiating sleep and/or difficulty maintaining sleep and/or waking up earlier than desired without being able to fall back to sleep; (b) sleep disturbance and associated daytime symptoms (of sleepiness) occurring at least three times/week; (c) symptoms have been present for at least 3 months; and (d) report of daytime impairment related to nighttime sleep difficulties. The following survey questions were used to assess the four criteria in each survey: (a) “Did you have trouble falling asleep?”, “Did you wake up several times a night?”, “Did you wake up earlier than planned?” and “Did you have trouble getting back to sleep after you woke up earlier than planned in the last 4 weeks?” (one possible answer: “yes”/”no”); (b) “How often per week did each problem occur?” [assessed independently for each of the questions in (a); one possible answer: “Less than once per week”; “1–2 times per week”; “3–4 times per week”; “More than 4 times per week”]; (c) “If you have a sleep problem how long have you been experiencing it?” [one possible answer: “Less than 3 months”; “More than 3 months”]; and (d) “How much do your sleep problems negatively affect your daily functioning?” [one possible answer: “Not at all”; “A little”; “Somewhat”; “Much”; “Very much”]. Chronic insomnia was present if criteria (a), (b) and (c) applied and if the question about effect on daily functioning was answered with “much” or “very much” (criterion d). In addition, we also considered chronic insomnia to be present if the participants reported having been diagnosed with chronic insomnia or circadian rhythm sleep disorder by a physician.

In addition, both surveys elicited information on sleep duration on weekdays and weekends (in hours and minutes), and this information was used to calculate an average daily sleep duration with the equation (5*sleep duration on a weekday + 2*sleep duration on weekend)/7. Short sleep was considered present if the average daily sleep was <6 h. Information on the chronotype was collected with the following question: “It is said that there are “morning” and “evening” types of people. Which one of these types do you consider yourself to be?” (one answer possible: “Early”; “Rather early”; “Rather late”; “Late”). Late chronotype was present if participants answered “late”. All sleep outcomes (chronic insomnia, short sleep and late chronotype) used for the analysis in both study samples (2017 and 2020) were treated as binary variables [Yes/No].

Additional outcome measures included optimism, happiness and health status. Dispositional optimism was measured with the LOT-R, a shorter, revised version of the original LOT [73] ranging from 0 to 24, with higher scores indicating higher optimism. The Subjective Happiness Scale [74] was used to assess current happiness, ranging from 0 to 20, with higher scores indicating higher happiness. The continuous variables (optimism, happiness) were then recoded into categorical variables with three categories (least, intermediate, most) based on the tertiles of each distribution. Finally, binary variables (least versus intermediate and most) were created for both outcomes. Information on health status was assessed with the question “In your opinion: How is your health status in general?”. Possible answers were “very good”, “good”, “intermediate”, “bad” and “very bad”. Based on this, a categorical variable with three categories (good, intermediate and bad) was created. Additionally, for the association analysis, a binary outcome variable was created (bad versus intermediate and good).

### 4.4. Confounder Assessment

The survey also collected information on the following potential confounders: age [2017: continuous, 2020: categories: <30; 30–39; 40–49; ≥50 years], sex (man; woman), education [elementary or high school; qualification for university entrance (Matura); university degree], work status (full-time employed; part-time employed; unemployed; retired; other), area of residence (urban; rural), marital status (single; married or in a partnership; divorced or widowed), children younger than 16 years of age to take care of (0; 1; 2; 3 or more), smoking status (current; ever; never), daily walking (on a weekday/weekend, continuous range in hours and minutes) and duration of moderate and vigorous physical activity (continuous, hours and minutes), in 2017, or weekly frequency of physical activity (at least 10 min) which increases heart or breathing rate (discrete, ranging 0–7 days) in 2020.

### 4.5. Statistical Analysis

We included 991 (2017) and 832 (2020) participants after excluding those with missing data in exposures and outcomes (excluded in 2017: n = 14; 2020: n = 168). The sociodemographic characteristics of both study groups are shown in Table 1, and Pearson’s Chi 2 tests were used to identify significant differences in TSO among the demographic variables. In Table 2, we showed the prevalence of our outcomes. For the main analyses, age- and multivariate-adjusted (MV) logistic regression models reporting odds ratios (OR) and 95% confidence intervals (95% CI) were calculated to evaluate the associations between exposures (daily average TSO in 2017 and 2020) and outcomes (chronic insomnia, short sleep, late chronotype, least optimistic tertile, least happy tertile and bad health status). For the multivariable-adjusted models, we considered a series of sociodemographic variables as confounders, a priori, based on the literature on known or suspected risk factors for the analyzed outcomes and the use of a Directed Acyclic Graph (DAG). Furthermore, we presented multivariable-adjusted models additionally adjusted for: (a) dispositional optimism and happiness; (b) physical activity; and (c) self-reported health status. In secondary analyses, we stratified by sex, age and area of residence. Interaction was tested with likelihood-ratio-tests, and *p*-values < 0.05 were considered as statistically significant. Generalized additive models (GAMs) with a smooth function were used to visualize the shape of the effects of TSO (continuous variable) on each outcome. We used natural splines (normal distribution) with 3 degrees of freedom and the identity link. We used age and multivariable adjusted models for potential confounders.

## 5. Conclusions

Our results provide evidence for the association between shorter TSO and higher prevalence of chronic insomnia, short sleep and late chronotype, as well as low optimism and happiness and poor health status in the general population, both before and during the pandemic. Altogether, special awareness campaigns and programs with recommendations on increasing TSO and exposure to daylight are needed to promote circadian hygiene and prevent sleep disorders, as well as mood and health impairments.

## Figures and Tables

**Table 1:** Study population characteristics by selected quintiles (q1, q3 and q5) of time spent outdoors (hours/day) in the 2017 and 2020 surveys.

	2017 Survey *				2020 Survey *			
	Total N = 991	TSOq1 0–1.7 h N = 203	TSOq3 2.7–3.7 h N = 209	TSOq5 5.1–12 h N = 198	Total N = 832	TSOq1 0–1 h N = 175	TSOq3 1.8–2.6 h N = 168	TSOq5 3.9–10.9 h N = 164
**Age categories**								
<25	129 (13.0%)	32 (15.8%)	30 (14.4%)	23 (11.6%)	101 (12.1%)	27 (15.4%)	18 (10.1%)	13 (7.9%)
25–34	180 (18.2%)	48 (23.6%)	38 (18.2%)	26 (13.1%)	149 (17.9%)	28 (16.0%)	47 (26.4%)	21 (12.8%)
35–44	231 (23.3%)	48 (23.6%)	49 (23.4%)	46 (23.2%)	192 (23.1%)	45 (25.7%)	41 (23.0%)	37 (22.6%)
45–54	249 (25.1%)	44 (21.7%)	53 (25.4%)	48 (24.2%)	202 (24.3%)	47 (26.9%)	35 (19.7%)	44 (26.8%)
>55	202 (20.4%)	31 (15.3%)	39 (18.7%)	55 (27.8%)	188 (22.6%)	28 (16.0%)	37 (20.8%)	49 (29.9%)
**Sex**								
Female	504 (50.9%)	112 (55.2%)	102 (48.8%)	88 (44.4%)	417 (50.1%)	88 (50.3%)	99 (55.6%)	73 (44.5%)
Male	487 (49.1%)	91 (44.8%)	107 (51.2%)	110 (55.6%)	415 (49.9%)	87 (49.7%)	79 (44.4%)	91 (55.5%)
**Education Level**								
Elementary or high school	402 (40.6%)	88 (43.3%)	71 (34.0%)	91 (46.0%)	305 (36.7%)	66 (37.7%)	57 (32.0%)	77 (47.0%)
University entry exam (Matura)	368 (37.1%)	66 (32.5%)	84 (40.2%)	75 (37.9%)	300 (36.1%)	65 (37.1%)	68 (38.2%)	49 (29.9%)
University degree	221 (22.3%)	49 (24.1%)	54 (25.8%)	32 (16.2%)	227 (27.3%)	44 (25.1%)	53 (29.8%)	38 (23.2%)
**Marital status**								
Single	298 (30.1%)	89 (43.8%)	59 (28.2%)	58 (29.3%)	275 (33.1%)	74 (42.3%)	58 (32.6%)	37 (22.6%)
Married/partnership	568 (57.3%)	96 (47.3%)	124 (59.3%)	114 (57.6%)	471 (56.6%)	83 (47.4%)	100 (56.2%)	106 (64.6%)
Divorced/widowed	125 (12.6%)	18 (8.9%)	26 (12.4%)	26 (13.1%)	86 (10.3%)	18 (10.3%)	20 (11.2%)	21 (12.8%)
**Parents of children <16 yrs**								
0	738 (74.5%)	162 (79.8%)	148 (70.8%)	143 (72.2%)	646 (77.6%)	148 (84.6%)	128 (71.9%)	120 (73.2%)
1	135 (13.6%)	20 (9.9%)	37 (17.7%)	28 (14.1%)	104 (12.5%)	17 (9.7%)	31 (17.4%)	21 (12.8%)
2	90 (9.1%)	18 (8.9%)	18 (8.6%)	17 (8.6%)	63 (7.6%)	8 (4.6%)	15 (8.4%)	15 (9.1%)
≥3	28 (2.8%)	3 (1.5%)	6 (2.9%)	10 (5.1%)	19 (2.3%)	2 (1.1%)	4 (2.2%)	8 (4.9%)
**Area of residence**								
Urban area	454 (45.8%)	104 (51.2%)	95 (45.5%)	75 (37.9%)	407 (48.9%)	115 (65.7%)	94 (52.8%)	63 (38.4%)
Rural area (<50.000 inhabitants)	410 (41.4%)	74 (36.5%)	89 (42.6%)	98 (49.5%)	303 (36.4%)	44 (25.1%)	68 (38.2%)	69 (42.1%)
Rural area (>50.000 inhabitants)	127 (12.8%)	25 (12.3%)	25 (12.0%)	25 (12.6%)	122 (14.7%)	16 (9.1%)	16 (9.0%)	32 (19.5%)
**Work status**								
Employed full time	522 (52.7%)	102 (50.2%)	119 (56.9%)	92 (46.5%)	432 (51.9%)	71 (40.6%)	101 (56.7%)	85 (51.8%)
Employed part time	110 (11.1%)	27 (13.3%)	20 (9.6%)	24 (12.1%)	106 (12.7%)	24 (13.7%)	24 (13.5%)	23 (14.0%)
Retired	124 (12.5%)	22 (10.8%)	19 (9.1%)	34 (17.2%)	93 (11.2%)	23 (13.1%)	12 (6.7%)	27 (16.5%)
Unemployed	61 (6.2%)	12 (5.9%)	14 (6.7%)	14 (7.1%)	74 (8.9%)	23 (13.1%)	14 (7.9%)	17 (10.4%)
*Other* **	174 (17.6%)	40 (19.7%)	37 (17.7%)	34 (17.2%)	127 (15.3%)	34 (19.4%)	27 (15.2%)	12 (7.3%)
**Night shift work**								
Never	618 (62.4%)	127 (62.6%)	134 (64.1%)	108 (54.5%)	496 (59.6%)	88 (50.3%)	111 (66.1%)	95 (57.9%)
Yes, in the past	255 (25.7%)	45 (22.2%)	56 (26.8%)	61 (30.8%)	264 (31.7%)	72 (41.1%)	46 (27.4%)	51 (31.1%)
Yes, currently	52 (5.2%)	15 (7.4%)	9 (4.3%)	15 (7.6%)	45 (5.4%)	7 (4.0%)	7 (4.2%)	13 (7.9%)
**Self-estimated health status**								
Good or very good	661 (66.7%)	120 (59.1%)	137 (65.6%)	147 (74.2%)	628 (75.5%)	114 (65.1%)	128 (71.9%)	133 (81.1%)
Intermediate	266 (26.8%)	60 (29.6%)	59 (28.2%)	41 (20.7%)	153 (18.4%)	38 (21.7%)	39 (21.9%)	28 (17.1%)
Bad or very bad	64 (6.5%)	23 (11.3%)	13 (6.2%)	10 (5.1%)	51 (6.1%)	23 (13.1%)	11 (6.2%)	3 (1.8%)
**Smoking status**								
Never	428 (43.2%)	105 (51.7%)	92 (44.0%)	78 (39.4%)	371 (44.6%)	77 (44.0%)	76 (42.7%)	62 (37.8%)
Former	269 (27.1%)	49 (24.1%)	53 (25.4%)	67 (33.8%)	220 (26.4%)	40 (22.9%)	44 (24.7%)	48 (29.3%)
Current	29 (29.7%)	49 (24.1%)	64 (30.6%)	53 (26.8%)	241 (29.0%)	58 (33.1%)	58 (32.6%)	54 (32.9%)
**Happiness scale; mean (SD)**	13.6 (3.4)	12.7 (3.5)	13.9 (3.2)	14.2 (3.5)	13.3 (3.4)	12.4 (3.4)	13.3 (3.3)	13.7 (3.5)
**Optimism index; mean (SD)**	14.3 (4.5)	13.0 (4.6)	14.7 (4.5)	15.0 (4.6)	13.9 (4.6)	12.6 (4.8)	14.0 (4.1)	14.2 (4.8)
**Physical activity; mean (SD)**								
Walking (hours/day)	1.3 (1.8)	0.8 (1.6)	1.2 (1.5)	1.8 (2.4)	0.8 (1.6)	0.5 (1.0)	0.9 (1.6)	1.2 (2.6)
Moderate physical activity (hours/day)	0.8 (1.6)	0.5 (1.0)	0.7 (1.2)	1.0 (1.7)				
Vigorous physical activity (hours/day)	1.0 (1.9)	0.7 (1.8)	0.9 (1.7)	1.1 (1.8)				
*Days per week with an activity (at least 10 min) that increases heart or breathing rate*					3.8 (2.4)	3.0 (2.1)	3.9 (2.3)	4.2 (2.6)

* 66 (6.7%) missing data entries for 2017; 27 (3.2%) missing data entries for 2020; ** Student/military/community service/unpaid work experience/disabled.

**Table 2:** Description of TSO and prevalence of sleep, mood and health outcomes in the 2017 and 2020 surveys.

	2017 Survey (N = 991)	2020 Survey (N = 832)
**TSO (median; IQR)**		
TSO weekday	2.0 (3.0)	2.0 (2.0)
TSO weekend	5.0 (4.0)	3.0 (3.0)
TSO daily average	3.1 (2.7)	2.1 (2.2)
**Sleep**		
Chronic insomnia N (%)	109 (11.0%)	109 (13.1%)
Short sleep N (%)	158 (15.9%)	126 (15.1%)
Late chronotype N (%)	200 (20.2%)	195 (23.4%)
**Mood and health**		
**Optimism score (0–24)**		
Median (IQR)	14 (6)	14 (6)
Mean (SD)	14.3 (4.5)	13.9 (4.6)
**Happiness score (0–20)**		
Median (IQR)	14 (4)	13 (5)
Mean (SD)	13.6 (3.4)	13.3(3.4)
**Least optimistic tertile N (%)**	359 (36.23%)	325 (39.1%)
**Least happy tertile N (%)**	342 (34.51%)	346 (41.6%)
**Bad health status N (%)**	64 (6.5%)	51 (6.1%)

**Table 3:** Association of time spent outdoors (TSO; hours/day) and prevalence of adverse sleep outcomes in 2017 and 2020.

	TSO-2017 Continuous *OR (95% CI)*	TSO-2020 Continuous *OR (95% CI)*
**Chronic Insomnia** N (n%)	109 [11.0%]	109 [13.1%]
Age adjusted	1.02 [0.93–1.12]	**0.79 [0.69–0.90]**
MV adjusted	1.01 [0.92–1.11]	**0.82 [0.71–0.94]**
MV adjusted + **opt.** + **happy**	1.06 [0.96–1.16]	**0.85 [0.74–0.98]**
MV adjusted + **activity**	0.98 [0.89–1.07]	**0.81 [0.70–0.93]**
MV adjusted + **health**	1.00 [0.92–1.10]	0.89 [0.78–1.03]
**Short sleep** N (%)	158 [15.9%]	126 [15.1%]
Age adjusted	**0.89 [0.82–0.97]**	**0.86 [0.77–0.97]**
MV adjusted	**0.88 [0.80–0.96]**	**0.89 [0.79–1.00]**
MV adjusted + **opt.** + **happy**	**0.89 [0.81–0.97]**	0.91 [0.80–1.02]
MV adjusted + **activity**	**0.88 [0.81–0.97]**	0.91 [0.81–1.02]
MV adjusted + **health**	**0.90 [0.82–0.98]**	0.89 [0.80–1.01]
**Late chronotype** N (%)	200 [20.2%]	195 [23.4%]
Age adjusted	**0.87 [0.81–0.95]**	**0.86 [0.78–0.95]**
MV adjusted	**0.87 [0.80–0.95]**	**0.87 [0.79–0.96]**
MV adjusted + **opt.** + **happy**	**0.88 [0.81–0.96]**	**0.89 [0.80–0.98]**
MV adjusted + **activity**	**0.90 [0.82–0.97]**	**0.81 [0.70–0.93]**
MV adjusted + **health**	**0.88 [0.81–0.96]**	**0.89 [0.80–0.98]**

**MV**: adjusted for age, gender, education, area, work status, marital status, children < 16 years, smoking status; **MV** + **opt.** + **happy**: additionally adjusted for optimism and happiness; **MV** + **activity**: additionally adjusted for daily walking and moderate and vigorous physical activity; **MV** + **health**: additionally adjusted for self-estimated health status.

**Table 4:** Association of time spent outdoors (TSO; hours/day) and happiness, optimism and health status in 2017 and 2020.

	TSO-2017 Continuous *OR (95%CI)*	TSO-2020 Continuous *OR (95%CI)*
**Least happy tertile**		
N (%)	342 [34.5%]	346 [41.6%]
Age adjusted	**0.89 [0.83–0.95]**	0.93 [0.86–1.00]
MV adjusted	**0.88 [0.83–0.95]**	0.94 [0.87–1.02]
MV adjusted + **opt.**	**0.91 [0.84–0.99]**	0.97 [0.88–1.07]
MV adjusted + **activity**	**0.89 [0.83–0.95]**	0.96 [0.88–1.04]
MV adjusted + **health**	**0.91 [0.85–0.98]**	0.98 [0.90–1.06]
**Least optimistic tertile**		
N (%)	359 [36.2%]	325 [39.1%]
Age adjusted	**0.93 [0.88–0.99]**	**0.89 [0.82–0.96]**
MV adjusted	**0.92 [0.87–0.98]**	**0.90 [0.83–0.98]**
MV adjusted + **happy**	0.98 [0.91–1.06]	0.92 [0.83–1.01]
MV adjusted + **activity**	**0.91 [0.85–0.97]**	0.92 [0.85–1.00]
MV adjusted + **health**	0.95 [0.89–1.01]	0.94 [0.86–1.02]
**Bad health status**		
N (%)	64 [6.5%]	51 [6.1%]
Age adjusted	**0.80 [0.69–0.92]**	**0.59 [0.46–0.75]**
MV adjusted	**0.80 [0.69–0.92]**	**0.62 [0.48–0.79]**
MV adjusted + **happy**	**0.83 [0.72–0.96]**	**0.63 [0.49–0.80]**
MV adjusted + **opt.**	**0.83 [0.72–0.96]**	**0.62 [0.48–0.79]**
MV adjusted+ **activity**	**0.81 [0.70–0.94]**	**0.64 [0.50–0.82]**

**MV**: adjusted for age, gender, education, area, work status, marital status, kids < 16 years, smoking status; **MV** + **opt.** + **happy**: additionally adjusted for optimism and happiness; **MV** + **activity**: additionally adjusted for daily walking and moderate and vigorous physical activity; **MV** + **health**: additionally adjusted for self-estimated health status.

## Data Availability

The data that support the findings of this study are available upon reasonable request.

## Supplementary Materials

The following supporting information can be downloaded at: https://www.mdpi.com/article/10.3390/clockssleep5030027/s1.
