# Supplementary material for: Time Spent Outdoors and Associations with Sleep, Optimism, Happiness and Health before and during the COVID-19 Pandemic in Austria
Source: Clocks Sleep. 2023 Jun 25;5(3):358–72. doi: 10.3390/clockssleep5030027 (PMC10366917; doi:10.3390/clockssleep5030027)
Supplement: Supplementary file 1 [file clockssleep-05-00027-s001.zip › clockssleep-2415496-supplementary.pdf]

Supplemental Materials

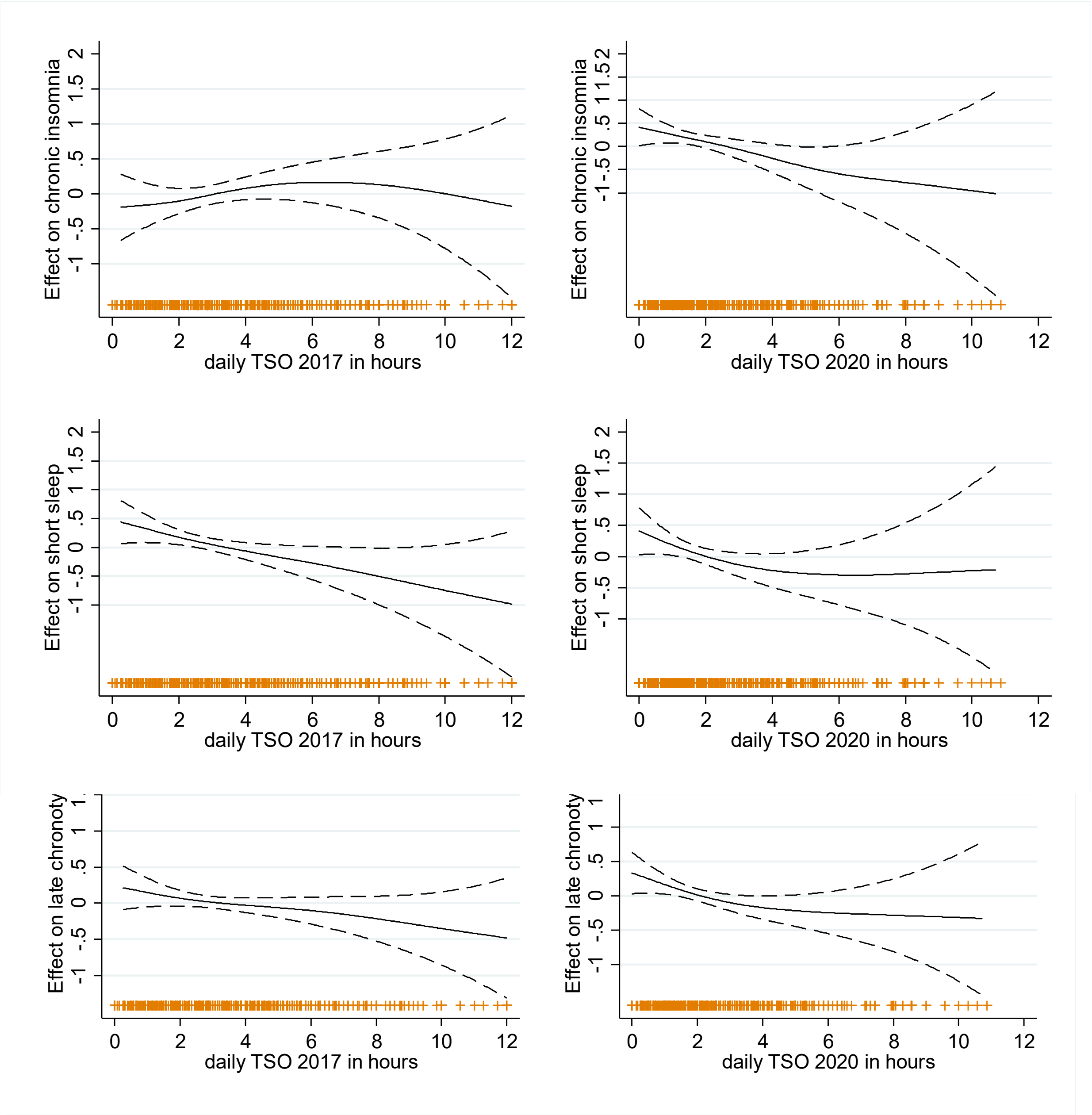

Supplemental Figure S1. Multivariable adjusted general additive models (GAM) splines (smooth function) for the association of time spent outdoors (hours/day) and sleep outcomes in the 2017 and 2020 survey.

Supplemental Table S1. Association of time spent outdoors (TSO) in quintiles (hours/day) and prevalence of adverse sleep outcomes in 2017

	**TSOq1 0-1.74h**	**TSOq2 1.75-2.71h**	**TSOq3 2.72-3.71h**	**TSOq4 3.72-5.11h**	**TSOq5 5.12-12h**
N (n%)	203 [20.5%]	194 [19.6%]	209 [21.1%]	187 [18.8%]	198 [20.0%]
**Chronic Insomnia**					
N (n%)	23 [11.3%]	11 [5.7%]	26 [12.4%]	25 [13.4%]	24 [12.1%]
Age adjusted OR (95%CI)	1	**0.46 [0.22, 0.98]**	1.10 [0.60,2.00]	1.18 [0.64,2.17]	1.05 [0.57,1.94]
MV adjusted OR (95%CI)	1	**0.45 [0.21,0.97]**	1.15 [0.62,2.14]	1.14 [0.61,2.14]	1.01 [0.54,1.89]
MV adjusted+ **opt.** + **happy** OR (95%CI)	1	0.55 [0.25,1.22]	1.56 [0.81,3.02]	1.51 [0.78,2.93]	1.44 [0.73,2.82]
MV adjusted+ **activity** OR (95%CI)	1	**0.45 [0.21,0.97]**	1.12 [0.60,2.09]	1.06 [0.56,2.01]	0.92 [0.48,1.76]
MV adjusted+ **health** OR (95%CI)	1	0.58 [0.25,1.33]	1.55 [0.79,3.06]	1.73 [0.87,3.46]	1.60 [0.80,3.21]
**Short sleep**					
N* (%)	45 [22.2%]	28 [14.4%]	34 [16.3%]	28 [15.0%]	23 [11.6%]
Age adjusted OR (95%CI)	1	**0.57 [0.34,0.97]**	**0.67 [0.41,1.09]**	**0.60 [0.35,1.01]**	**0.44 [0.25,0.76]**
MV adjusted OR (95%CI)	1	**0.49 [0.29,0.85]**	**0.59 [0.35,0.99]**	**0.52 [0.30,0.89]**	**0.38 [0.22,0.68]**
MV adjusted+ **opt.** + **happy** OR (95%CI)	1	**0.53 [0.31,0.91]**	0.64 [0.38,1.07]	**0.54 [0.31,0.94]**	**0.43 [0.24,0.76]**
MV adjusted + **activity** OR (95%CI)	1	**0.48 [0.28,0.83]**	**0.58 [0.35,0.97]**	**0.49 [0.28,0.85]**	**0.37 [0.20,0.65]**
MV adjusted+ **health** OR (95%CI)	1	**0.52 [0.30,0.89]**	**0.60 [0.36,1.01]**	**0.55 [0.32,0.95]**	**0.43 [0.24,0.77]**
**Late chronotype**					
N* (%)	50 [24.6%]	41 [21.1%]	46 [22.0%]	36 [19.3%]	27 [13.6%]
Age OR (95%CI)	1	0.83 [0.52,1.32]	0.87 [0.55,1.37]	0.74 [0.45,1.19]	**0.49 [0.29,0.82]**
MV OR (95%CI)	1	0.78 [0.48,1.27]	0.87 [0.54,1.40]	0.70 [0.42,1.17]	**0.49 [0.29,0.83]**
MV + **opt.** + **happy** OR (95%CI)	1	0.82 [0.50,1.34]	0.93 [0.57,1.50]	0.74 [0.44,1.23]	**0.52 [0.30,0.90]**
MV + **activity** OR (95%CI)	1	0.85 [0.52,1.40]	0.95 [0.59,1.54]	0.82 [0.48,1.38]	**0.57 [0.33,0.98]**
MV + **health** OR (95%CI)	1	0.81 [0.49,1.33]	0.90 [0.56,1.46]	0.74 [0.44,1.23]	**0.51 [0.30,0.88]**

MV: adjusted for age, gender, education, area, workstatus, marital, kids <16 years, smoking status

MV + opt. + happy: adjusted for MV + optimism and happiness

MV + activity: adjusted for MV + daily walking and moderate and vigorous physical activity

MV + health: adjusted for MV + self-estimated health status

Supplemental Table S2. Association of time spent outdoors (TSO) in quintiles and prevalence of adverse sleep outcomes in 2020

	**TSOq1 0-1.00h**	**TSOq2 1.01-1.79h**	**TSOq3 1.80-2.57h**	**TSOq4 2.58-3.86h**	**TSOq5 3.87-10.86h**
N (n%)	175 [21.0%]	1163 [19.6%]	168 [20.2%]	162 [19.5%]	164 [19.7%]
**Chronic Insomnia**					
N (n%)	36 [20.6%]	18 [11.0%]	25 [14.9%]	18 [11.1%]	12 [7.3%]
Age adjusted OR (95%CI)	1	**0.48 [0.26,0.89]**	0.68 [0.39,1.20]	**0.46 [0.25,0.85]**	**0.29 [0.15,0.59]**
MV adjusted OR (95%CI)	1	0.57 [0.30,1.07]	0.76 [0.42,1.39]	0.56 [0.29,1.09]	**0.34 [0.16,0.69]**
MV adjusted+ **opt. + happy** OR (95%CI)	1	0.75 [0.37,1.51]	1.03 [0.54,1.98]	0.77 [0.38,1.57]	**0.39 [0.18,0.86]**
MV adjusted+ **activity** OR (95%CI)	1	0.55 [0.29,1.05]	0.75 [0.41,1.37]	0.55 [0.28,1.08]	**0.32 [0.15,0.68]**
MV adjusted + **health** OR (95%CI)	1	0.71 [0.36,1.40]	0.82 [0.43,1.56]	0.83 [0.41,1.68]	0.51 [0.24,1.10]
**Short sleep**					
N (%)	41 [23.4%]	22 [13.5%]	22 [13.1%]	20 [12.4%]	21 [12.8%]
Age adjusted OR (95%CI)	1	**0.51 [0.28,0.90]**	**0.48 [0.27,0.85]**	**0.43 [0.24,0.78]**	**0.47 [0.26,0.84]**
MV adjusted OR (95%CI)	1	0.61 [0.33,1.11]	0.57 [0.31,1.05]	0.54 [0.29,1.02]	**0.52 [0.28,0.96]**
MV adjusted + **opt. + happy** OR (95%CI)	1	0.67 [0.37,1.24]	0.60 [0.32,1.12]	0.62 [0.33,1.18]	0.57 [0.30,1.06]
MV adjusted + **activity** OR (95%CI)	1	0.66 [0.36,1.23]	0.63 [0.34,1.16]	0.61 [0.32,1.17]	0.59 [0.31,1.12]
MV adjusted + **health** OR (95%CI)	1	0.62 [0.34,1.15]	0.58 [0.31,1.08]	0.56 [0.29,1.06]	0.54 [0.29,1.02]
**Late chronotype**					
N (%)	62 [35.4%]	32 [19.6%]	45 [26.8%]	24 [14.8%]	32 [19.5%]
Age adjusted OR (95%CI)	1	**0.44 [0.27,0.73]**	0.67 [0.42,1.06]	**0.31 [0.18,0.53]**	**0.44 [0.27,0.73]**
MV adjusted OR (95%CI)	1	**0.48 [0.28,0.80]**	0.71 [0.43,1.16]	**0.32 [0.18,0.56]**	**0.45 [0.27,0.76]**
MV adjusted + **opt. + happy** OR (95%CI)	1	**0.52 [0.31,0.89]**	0.78 [0.47,1.29]	**0.36 [0.20,0.65]**	**0.49 [0.29,0.84]**
MV adjusted + **activity** OR (95%CI)	1	**0.53 [0.31,0.89]**	0.79 [0.48,1.30]	**0.37 [0.21,0.66]**	**0.53 [0.31,0.91]**
MV adjusted + **health** OR (95%CI)	1	**0.50 [0.30,0.84]**	0.72 [0.44,1.19]	**0.35 [0.20,0.62]**	**0.49 [0.29,0.85]**

MV: adjusted for age, gender, education, area, workstatus, marital, kids <16 years, smoking status

MV + opt. + happy: adjusted for MV + optimism and happiness

MV + activity: adjusted for MV + daily walking and moderate and vigorous physical activity

MV + health: adjusted for MV + self-estimated health status

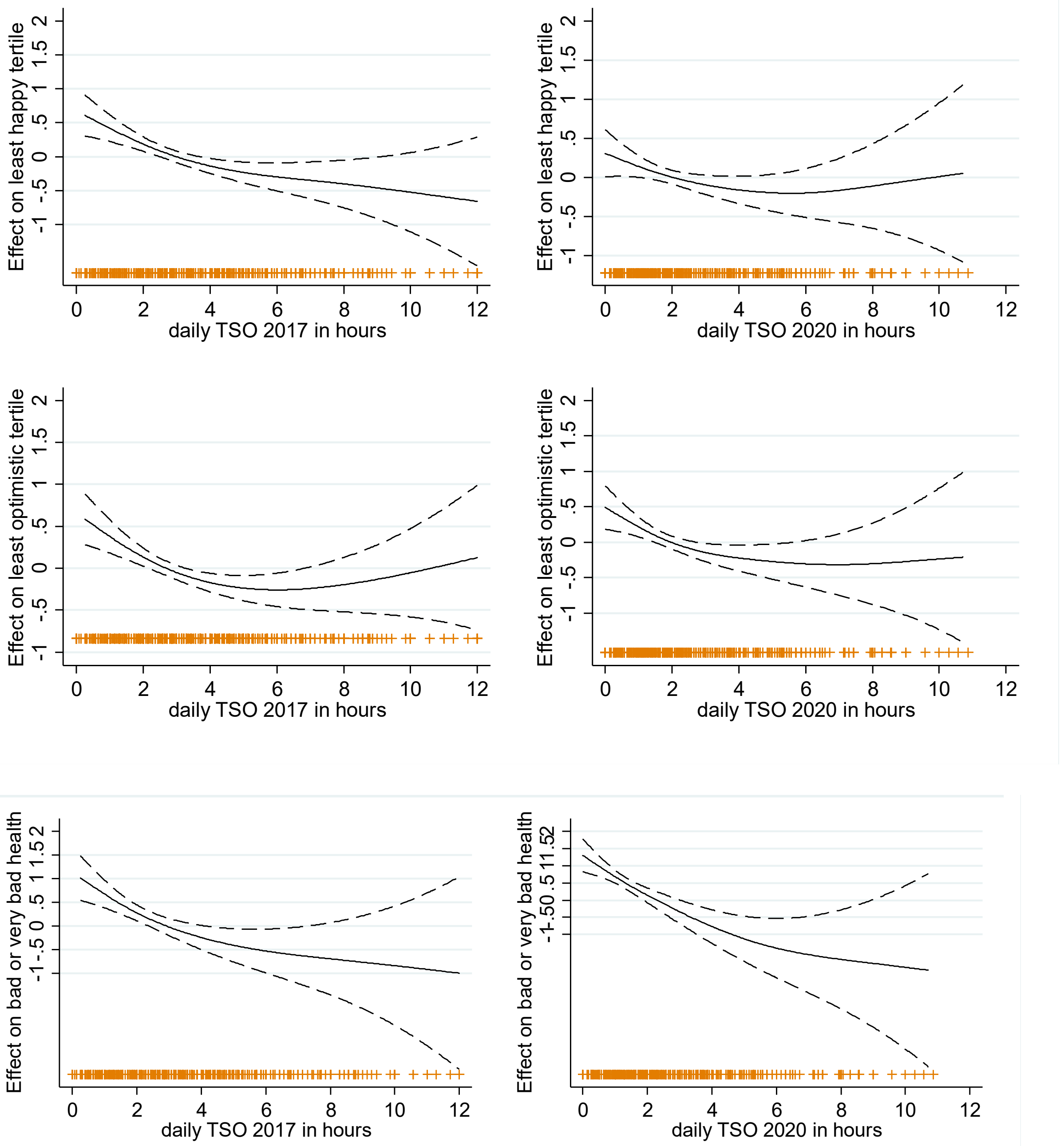

Supplemental Figure S2. Multivariable adjusted general additive models (GAM) splines (smooth function) for the association of time spent outdoors (hours/day) and optimism, happiness and health status in the 2017 and 2020 survey.

Supplemental Table S3. Association of time spent outdoors (TSO) in quintiles (hours/day) and prevalence of least happy tertile, least optimistic tertile and bad health status in 2017

	**TSOq1 0-1.74h**	**TSOq2 1.75-2.71h**	**TSOq3 2.72-3.71h**	**TSOq4 3.72-5.11h**	**TSOq5 5.12-12h**
N (n%)	203 [20.5%]	194 [19.6%]	209 [21.1%]	187 [18.8%]	198 [20.0%]
**Least happy tertile**					
N (n%)	95 [46.8%]	67 [34.5%]	62 [29.7%]	63 [33.7%]	55 [27.2%]
Age adjusted OR (95%CI)	1	**0.63 [0.42,0.95**	**0.50 [0.33,0.75]**	**0.61 [0.41,0.93]**	**0.47 [0.31,0.72]**
MV adjusted OR (95%CI)	1	**0.64 [0.42,0.98]**	**0.50 [0.33,0.76]**	**0.62 [0.40,0.94]**	**0.45 [0.29,0.69]**
MV adjusted + **opt.** OR (95%CI)	1	0.81 [0.48,1.35]	**0.59 [0.35,0.98]**	0.71 [0.42,1.20]	0.61 [0.36,1.03]
MV adjusted + **activity** OR (95%CI)	1	0.67 [0.44,1.03]	**0.51 [0.33,0.77]**	**0.65 [0.42,1.00]**	**0.47 [0.30,0.73]**
MV adjusted + **health** OR (95%CI)	1	0.72 [0.46,1.11]	**0.52 [0.34,0.81]**	0.70 [0.45,1.09]	**0.53 [0.34,0.83]**
**Least optimistic tertile**					
N (%)	100 [49.3%]	65 [33.5%]	68 [32.5%]	64 [34.2%]	62 [31.3%]
Age adjusted OR (95%CI)	1	**0.54 [0.36,0.82]**	**0.51 [0.34,0.77]**	**0.57 [0.37,0.86]**	**0.51 [0.34,0.77]**
MV adjusted OR (95%CI)	1	**0.58 [0.38,0.88]**	**0.54 [0.36,0.83]**	**0.57 [0.37,0.87]**	**0.48 [0.32,0.74]**
MV adjusted + **happy** OR (95%CI)	1	0.63 [0.38,1.06]	0.71 [0.43,1.19]	0.67 [0.40,1.13]	0.67 [0.40,1.12]
MV adjusted + **activity** OR (95%CI)	1	**0.57 [0.37,0.87]**	**0.53 [0.35,0.80]**	**0.54 [0.35,0.83]**	**0.45 [0.29,0.69]**
MV adjusted + **health** OR (95%CI)	1	**0.63 [0.41,0.97]**	**0.58 [0.38,0.88]**	**0.63 [0.41,0.98]**	**0.56 [0.36,0.86]**
**Bad or very bad health status**					
N (%)	23 [11.3%]	10 [5.2%]	13 [6.2%]	8 [4.3%]	10 [5.1%]
Age adjusted OR (95%CI)	1	**0.40 [0.18,0.86]**	0.49 [0.24,1.00]	**0.32 [0.14,0.74]**	**0.37 [0.17,0.81]**
MV adjusted OR (95%CI)	1	**0.37 [0.16,0.83]**	**0.47 [0.22,0.99]**	**0.30 [0.13,0.72]**	**0.34 [0.15,0.76]**
MV adjusted + **happy** OR (95%CI)	1	0.47 [0.21,1.08]	0.61 [0.28,1.34]	**0.37 [0.15,0.91]**	**0.40 [0.17,0.94]**
MV adjusted+ **opt.** OR (95%CI)	1	0.50 [0.22,1.17]	0.63 [0.28,1.40]	**0.34 [0.13,0.86]**	**0.40 [0.17,0.96]**
MV adjusted + **activity** OR (95%CI)	1	**0.40 [0.18,0.91]**	0.48 [0.22,1.02]	**0.31 [0.13,0.75]**	**0.37 [0.16,0.85]**

MV: adjusted for age, gender, education, area, workstatus, marital, kids <16 years, smoking status, optimism index

MV + opt. + happy: adjusted for MV + optimism and happiness

MV + activity: adjusted for MV + daily walking and moderate and vigorous physical activity

MV + health: adjusted for MV + self-estimated health status

Supplemental Table S4. Association of time spent outdoors (TSO) in quintiles and prevalence of least happy tertile, least optimistic tertile and bad health status in 2020

	TSOq1 0-1.74h	TSOq2 1.75-2.71h	TSOq3 2.72-3.71h	TSOq4 3.72-5.11h	TSOq5 5.12-12h
N (n%)	175 [21.0%]	1163 [19.6%]	168 [20.2%]	162 [19.5%]	164 [19.7%]
**Least happy tertile**					
N (n%)	91 [52.0%]	63 [38.7%]	71 [42.3%]	60 [37.0%]	61 [37.2%]
Age adjusted OR (95%CI)	1	**0.57 [0.37,0.89]**	0.66 [0.43,1.02]	**0.56 [0.36,0.87]**	**0.58 [0.37,0.90]**
MV adjusted OR (95%CI)	1	**0.61 [0.39,0.95]**	0.72 [0.46,1.13]	**0.59 [0.37,0.93]**	**0.61 [0.38,0.96]**
MV adjusted+ **opt.** OR (95%CI)	1	0.80 [0.46,1.41]	0.96 [0.55,1.66]	0.83 [0.47,1.47]	0.68 [0.39,1.21]
MV adjusted+ **activity** OR (95%CI)	1	0.65 [0.41,1.03]	0.77 [0.49,1.22]	0.65 [0.41,1.05]	0.68 [0.42,1.09]
MV adjusted+ **health** OR (95%CI)	1	0.67 [0.42,1.07]	0.75 [0.47,1.19]	0.72 [0.45,1.17]	0.74 [0.46,1.19]
**Least optimistic tertile**					
N (%)	98 [56.0%]	55 [33.7%]	62 [36.9%]	51 [31.5%]	59 [36.0%]
Age adjusted OR (95%CI)	1	**0.40 [0.25,0.62]**	**0.47 [0.30,0.72]**	**0.38 [0.24,0.59]**	**0.47 [0.30,0.73]**
MV adjusted OR (95%CI)	1	**0.42 [0.26,0.67]**	**0.50 [0.32,0.80]**	**0.40 [0.25,0.65]**	**0.51 [0.32,0.82]**
MV adjusted + **happy** OR (95%CI)	1	**0.50 [0.29,0.87]**	**0.53 [0.31,0.91]**	**0.46 [0.26,0.81]**	0.60 [0.35,1.05]
MV adjusted+ **activity** OR (95%CI)	1	**0.45 [0.28,0.73]**	**0.54 [0.34,0.86]**	**0.45 [0.28,0.74]**	**0.58 [0.36,0.94]**
MV adjusted+ **health** OR (95%CI)	1	**0.46 [0.28,0.74]**	**0.51 [0.31,0.82]**	**0.49 [0.30,0.81]**	0.62 [0.38,1.01]
**Bad or very bad health status**					
N (%)					
Age adjusted OR (95%CI)	1	**0.33 [0.14,0.78]**	**0.46 [0.21,0.98]**	**0.21 [0.08,0.55]**	**0.10 [0.03,0.35]**
MV adjusted OR (95%CI)	1	0.42 [0.17,1.05]	0.66 [0.29,1.54]	**0.26 [0.09,0.74]**	**0.10 [0.03,0.36]**
MV adjusted + **happy** OR (95%CI)	1	0.52 [0.20,1.34]	0.76 [0.31,1.83]	**0.28 [0.10,0.82]**	**0.11 [0.03,0.41]**
MV adjusted+ **opt.** OR (95%CI)	1	0.47 [0.17,1.29]	0.88 [0.35,2.16]	**0.30 [0.10,0.92]**	**0.08 [0.02,0.33]**
MV adjusted+ **activity** OR (95%CI)	1	0.46 [0.18,1.17]	0.72 [0.30,1.70]	0.31 [0.11,0.89]	0.11 [0.03,0.41]

MV: adjusted for age, gender, education, area, workstatus, marital, kids <16 years, smoking status

MV + opt. + happy: adjusted for MV + optimism and happiness

MV + activity: adjusted for MV + daily walking and moderate and vigorous physical activity

MV + health: adjusted for MV + self-estimated health status

Supplemental Table S5. Association of time spent outdoors (TSO) (hours/day) and prevalence of all outcomes in 2017 and 2020 stratified by sex.

	**TSO-2017 continuous Per units increase**			**TSO-2020 continuous Per units increase**		
	Total	Female	Male	Total	Female	Male
**N(n%)**	991	504 [50.9%]	487 [49.1%]	832	417 [50.1%]	415 [49.9%]
**Chronic Insomnia**						
MV OR (95%CI)	1.01 [0.92-1.11]	1.08 [0.94-1.24]	0.96 [0.84-1.10]	**0.82 [0.71-0.94]**	0.88 [0.74,1.06]	**0.73 [0.58-0.92]**
Interaction (p-value)	0.734			0.497		
**Short sleep**						
MV OR (95%CI)	**0.88 [0.80-0.96]**	0.80 [0.68-0.93]	0.94 [0.84-1.05]	**0.89 [0.79-1.00]**	0.89 [0.73,1.08]	0.88 [0.76-1.02]
Interaction (p-value)	0.073			0.568		
**Late chronotype**						
MV OR (95%CI)	**0.87 [0.80-0.95]**	0.98 [0.87-1.10]	**0.77 [0.68-0.88]**	**0.87 [0.79-0.96]**	**0.82 [0.69,0.96]**	0.91 [0.80-1.04]
Interaction (p-value)	**0.038**			0.307		
**Least happiness tertile**						
MV OR (95%CI)	**0.88 [0.83-0.95]**	**0.86 [0.78-0.96]**	**0.89 [0.81-0.97]**	0.94 [0.87-1.02	1.02 [0.91,1.15]	**0.87 [0.77-0.97]**
Interaction (p-value)	0.644			0.165		
**Least optimism tertile**						
MV OR (95%CI)	**0.92 [0.87-0.98]**	**0.89 [0.80-0.98]**	0.95 [0.88-1.04]	**0.90 [0.83-0.98]**	0.93 [0.82-1.05]	**0.86 [0.76-0.97]**
Interaction (p-value)	0.30			0.822		
**Bad or very bad health status**						
MV OR (95%CI)	**0.80 [0.69-0.92]**	**0.70 [0.55-0.91]**	0.84 [0.70-1.00] P=0.0055	**0.62 [0.48-0.79]**	**0.72 [0.52-0.99]**	0.47 [0.32-0.71]
Interaction (p-value)	0.067			0.776		

MV: adjusted for age, gender, education, area, workstatus, marital, kids <16 years, smoking status

MV + opt. + happy: adjusted for MV + optimism and happiness

MV + activity: adjusted for MV + daily walking and moderate and vigorous physical activity

MV + health: adjusted for MV + self-estimated health status
